# Medical care costs according to the stage and subtype of breast cancer in a municipal setting: a case study of Hachioji City, Japan

**DOI:** 10.1007/s12282-023-01517-7

**Published:** 2023-11-20

**Authors:** Yoshitaka Nishikawa, Nobukazu Agatsuma, Takahiro Utsumi, Taro Funakoshi, Yukiko Mori, Yuki Nakamura, Nobuaki Hoshino, Takahiro Horimatsu, Takumi Saito, Soichiro Kashihara, Jun Fukuyoshi, Rei Goto, Masakazu Toi, Yoshimitsu Takahashi, Takeo Nakayama

**Affiliations:** 1 Department of Health Informatics, Kyoto University School of Public Health, Yoshidakonoecho, Sakyo-Ku, Kyoto, 606-8501 Japan; 2 Department of Gastroenterology and Hepatology, Kyoto University Graduate School of Medicine, Kyoto, Japan; 3 Department of Therapeutic Oncology, Kyoto University Graduate School of Medicine, Kyoto, Japan; 4 Department of Breast Surgery, Kyoto University Graduate School of Medicine, Kyoto, Japan; 5 Institute for Advancement of Clinical and Translational Science (iACT), Kyoto University Hospital, Kyoto, Japan; 6 Department of Surgery, Kyoto University Graduate School of Medicine, Kyoto, Japan; 7 Cancerscan, Co., Ltd, Tokyo, Japan; 8 Graduate School of Business Administration, Keio University, Yokohama, Japan; 9 Tokyo Metropolitan Cancer and Infectious Disease Center Komagome Hospital, Tokyo, Japan

**Keywords:** Breast neoplasms, Medical care costs, Healthcare administrative claims, Case study

## Abstract

**Background:**

It is important to assess whether the early detection of breast cancer affects medical care costs. However, research remains scant on the actual medical care costs associated with breast cancer treatment in Japan. This study aimed to determine the medical care costs of breast cancer treatment based on its stage using national health insurance claims data.

**Methods:**

This was an observational study including patients with breast cancer who had undergone breast cancer treatment, as defined by the disease name and related treatment codes. Between August 2013 and June 2016, patients who underwent surgical treatment without axillary lymph node dissection and other radical treatment were classified as the curable group, while those who underwent palliative treatment were classified as the non-curable group. Patients were further stratified by subtype. The total and treatment-specific medical care costs for the five years were calculated using the national health insurance claims data of Hachioji City between August 2013 and May 2021.

**Results:**

The mean total medical care costs for the curable and non-curable groups for the 5 years were JPY 3958 thousand (standard deviation 2664) and JPY 8289 thousand (8482), respectively. The mean medical care costs for specific breast cancer treatment for the curable and non-curable groups were JPY 1142 (728) thousand and JPY 3651 thousand (5337), respectively. Further, human epidermal growth factor receptor 2 + , Hormone + patients had the highest mean cost over the 5 years.

**Conclusions:**

The results suggest that the early detection of breast cancer may reduce medical care costs at the patient level.

**Supplementary Information:**

The online version contains supplementary material available at 10.1007/s12282-023-01517-7.

## Introduction

Breast cancer is the most commonly diagnosed cancer and leading cause of death in women [1]. Breast cancer screening using mammography, which reduces mortality from the disease [2], is conducted in women aged 40 years or older as per Japan’s public health policy [3]. For mammogram-positive patients, it is important to increase the cancer screening rate via mammography and subsequent diagnostic investigations such as ultrasound and magnetic resonance imaging [4, 5]. Approximately 1,700 municipalities in Japan are responsible for providing and financing population-based cancer screening programs for their citizens [3]. Local municipalities are thus keenly interested in the budgetary impact of cancer screening, namely, the costs of both screening and related medical care. However, little information is available on whether the early detection of breast cancer reduces medical care costs in Japan.

To assess the efficacy of the early detection of breast cancer in reducing medical care costs, it is important to determine these costs based on the breast cancer stage. Some reports have described medical care costs for breast cancer in certain stages and periods [6, 7]. In Japan, first-year medical care costs have been reported using Diagnosis Procedure Combination data [8]. However, these data are based on hospital-specific records and fail to capture patients’ transitions between medical institutions. Moreover, treatment costs for breast cancer can vary based on both the disease characteristics such as hormone receptor and human epidermal growth factor receptor 2 (HER2) status [9, 10] and patient-specific factors such as complications and comorbidities [11]. Therefore, it is crucial to capture both the total and the treatment-specific costs associated with breast cancer treatment across medical institutions over an extended period.

For health insurers such as Japanese municipalities, the total medical care costs of breast cancer treatment may hold more significance than treatment-specific costs. Although municipalities are responsible for population-based cancer screening in Japan [3], a comprehensive analysis of the total and treatment-specific costs for patients with breast cancer for an extended period, stratified by stage and subtype, is lacking. Thus, this study aimed to determine the total and treatment-specific medical care costs of treating breast cancer by stage and subtype in Japan. It used national health insurance claims data, which provide a complete view of patient treatment trajectories across healthcare institutions. Its findings will help health insurers understand the financial burden of breast cancer. Furthermore, it is the first study in Japan to examine the medical care costs of breast cancer patients using a municipality’s national health insurance claims data.

## Materials and methods

### Design and setting

In this observational study, we used anonymized national health insurance claims data (inpatient, outpatient, and dispensing pharmacy) from May 2013 to May 2021 in Hachioji City, located in the west of Tokyo. As of 2020, Hachioji City had approximately 580,000 residents covered by 35 hospitals, including two designated cancer hospitals. Our working group was commissioned to analyze medical care costs in Hachioji City as part of the city’s cancer screening project [12].

### Patients

This study included female patients with breast cancer or C50 according to the International Classification of Diseases, Tenth Revision, between May 2013 and June 2016. We also included patients with codes for specific breast cancer treatment between August 2013 and June 2016. We created a study group comprising two breast surgery specialists accredited by the Japanese Breast Cancer Society, two clinical oncologists certified by the Japanese Society of Medical Oncology, two general clinical oncologists from the Japan Board of Cancer Therapy, and two oncologists with substantial experience in analyzing national health insurance claims data. This group extracted the procedure codes associated with certain breast cancer procedures from the Ministry of Health, Labour and Welfare and the codes for the anticancer agents insurance covered for treating breast cancer, as shown in Table 1. Our previous study identified patients with breast cancer from national health insurance claims data [13]. We used an algorithm that defined treatment using the aforementioned codes for specific breast cancer treatment: surgery, radiation, chemotherapy, antibody therapy, and hormone therapy (Table 1).

**Table 1:** The codes associated with specific procedures for breast cancer, and the claim computer processing system codes of the anticancer agents for breast cancer

Specific breast cancer treatment	Procedure code
Surgical treatment for malignant breast tumor with axillary lymph node dissection	
Extended mastectomy	150,121,910
Partial mastectomy	150,262,710
Areola-preserving breast-conserving surgery	150,386,510
With bilateral axillary lymph node dissection	150,122,150
Surgical treatment for malignant breast tumor without axillary lymph node dissection	
Mastectomy	150,316,510
Partial mastectomy	150,303,110
Areola-preserving breast-conserving surgery	150,386,410
Surgical treatment for malignant breast tumor	
Cryoablation	150,121,550
Simple mastectomy	150,121,610
Mastectomy without pectoralis muscle excision	150,121,710
Mastectomy with pectoralis muscle excision	150,121,810
Sentinel lymph node biopsy	150,345,870, 150,345,970
Lymph node dissection	
Axillary lymph nodes	150,156,610
Supra/ infraclavicular lymph nodes	150,156,510
Parasternal lymph nodes	150,156,710
Other surgical treatment (not limited to malignant breast tumor)	
Breast tumorectomy	150,121,110, 150,121,210, 190,179,610, 190,179,710
Mastectomy	150,121,410, 150,413,710
Segmental mastectomy	150,274,610
Breast reconstructive surgery	150,292,310, 150,316,610, 150,316,710, 150,369,750, 150,369,850, 150,371,710, 150,371,910, 150,374,010
Incision and drainage of breast abscess	150,120,910
Radiotherapy	
X-ray therapy	180,008,810, 180,019,410
High-energy radiotherapy	180,020,710, 180,020,810, 180,020,910, 180,021,010, 180,021,110, 180,021,210, 180,021,310, 180,021,410, 180,021,510, 180,021,610, 180,021,710, 180,021,810, 180,021,910, 180,022,010
Intensity modulated radiation therapy	180,031,710, 180,031,910, 180,032,010
Whole breast radiation therapy	180,043,270
Chemotherapy	
Generic name	Claim computer processing system code
Cytotoxic anticancer treatment	
Methotrexate	620,007,515, 622,221,301, 644,210,048, 644,210,049
Cytarabine	620,003,713
Capecitabine	610,470,009, 622,656,401, 622,674,301, 622,677,701, 622,679,001, 622,695,801, 622,700,101
Fluorouracil	610,461,237, 614,210,003, 614,210,004, 614,220,008, 614,220,009, 622,047,901, 622,229,101, 622,412,501, 622,412,601, 640,463,105
Uracil-tegafur	620,915,001, 621,929,901, 621,930,001, 621,930,101
Oteracil potassium, gimeracil, tegafur	620,009,353, 620,009,354, 620,915,501, 620,915,601, 622,243,001, 622,243,101, 622,254,901, 622,255,001, 622,256,001, 622,256,101, 622,266,701, 622,266,801, 622,275,701, 622,275,801, 622,285,701, 622,285,801, 622,294,601, 622,294,701, 622,397,101, 622,397,201, 622,397,301, 622,397,401, 622,430,801, 622,430,901, 622,434,701, 622,434,801, 622,487,301, 622,487,401, 622,497,901, 622,498,001, 622,537,501, 622,537,601
Tegafur	610,461,179, 620,004,566, 620,004,820, 620,005,087, 620,006,168, 620,906,901, 620,907,005, 620,910,101
Doxifluridine	614,210,128, 614,210,129
Gemcitabine hydrochloride	621,970,201, 621,970,202, 621,970,301, 621,970,302, 621,973,401, 621,973,501, 621,994,401, 621,994,501, 622,019,601, 622,019,701, 622,028,601, 622,028,701, 622,062,103, 622,062,105, 622,062,203, 622,062,205, 622,098,901, 622,099,001, 622,202,401, 622,202,501, 622,272,801, 622,272,901, 622,393,001, 622,393,101, 622,460,401, 622,460,501, 622,487,701, 622,487,801, 640,454,012, 640,454,013
Doxorubicin hydrochloride	620,003,675, 620,004,851, 621,983,201, 621,983,301, 621,995,301, 621,995,401, 622,014,001
Epirubicin hydrochloride	620,003,790, 620,003,791, 620,003,792, 620,003,793, 620,007,224, 620,007,225, 620,008,174, 620,008,175, 620,009,523, 620,009,524, 620,009,525, 620,009,526, 620,009,527, 621,966,401, 621,966,501, 621,966,601, 621,966,701, 622,246,601, 622,246,701, 622,760,200, 622,760,300, 622,760,400
Mitoxantrone hydrochloride	640,454,032, 644,290,005
Pirarubicin	620,003,762, 620,003,763, 620,005,206, 620,005,207, 622,513,101
Aclarubicin hydrochloride	620,005,148
Paclitaxel	620,003,751, 620,003,752, 620,004,170, 620,004,171, 620,005,688, 620,005,689, 620,005,690, 621,970,101, 622,009,101, 622,009,102, 622,009,201, 622,009,202, 622,082,001, 622,082,101, 622,259,101, 622,259,201, 622,375,001, 622,375,101, 622,760,500, 622,760,600, 622,760,700
Docetaxel hydrate	620,919,801, 620,919,901, 622,068,501, 622,068,601, 622,215,301, 622,215,401, 622,231,801, 622,231,901, 622,272,001, 622,272,101, 622,283,101, 622,283,201, 622,285,201, 622,285,301, 622,285,401, 622,290,401, 622,290,501, 622,294,901, 622,295,001, 622,295,501, 622,295,601, 622,354,801, 622,354,901, 622,356,401, 622,356,501, 622,408,501, 622,408,601, 622,417,601, 622,417,701, 622,429,301, 622,429,401, 622,435,002, 622,435,102
Eribulin mesylate	622,085,201
Vinorelbine tartrate	621,954,401, 621,954,501, 640,432,004, 640,432,005
Carboplatin	620,004,117, 620,004,118, 620,004,119, 620,004,120, 620,004,121, 620,004,122, 620,004,732, 620,004,733, 620,004,734, 620,007,254, 620,007,255, 620,007,256, 621,754,502, 621,754,602, 621,754,702, 622,098,103, 622,098,203, 622,098,303, 622,761,100, 622,761,200, 622,761,300
Cisplatin	620,004,129, 620,004,130, 620,004,131, 620,006,298, 620,006,299, 620,006,300, 620,008,946, 620,008,947, 620,008,948, 620,009,545, 620,009,546, 620,009,547, 620,923,202, 620,923,301, 620,923,602, 620,923,701, 620,924,002, 620,924,101, 622,760,800, 622,760,900, 622,761,000, 644,290,002, 644,290,003, 644,290,004
Cyclophosphamide hydrate	620,005,941, 622,181,601, 640,453,101, 644,210,037
Mitomycin C	620,000,328, 620,000,329
Irinotecan hydrochloride hydrate	620,007,257, 620,007,258, 620,009,515, 620,009,516, 620,009,517, 620,009,518, 620,009,519, 620,009,520, 620,009,521, 620,009,522, 620,919,501, 620,919,701, 621,900,302, 621,900,402, 622,019,401, 622,019,501, 622,059,701, 622,059,801, 622,091,101, 622,091,201, 622,230,201, 622,230,301, 622,236,901, 622,237,001, 622,258,901, 622,259,001, 622,470,401, 622,470,501
Nogitecan hydrochloride	620,005,197
Monoclonal antibody treatment	
Bevacizumab	620,004,872, 620,004,873
Lapatinib tosilate hydrate	621,911,601
Pazopanib hydrochloride	622,201,801
Pertuzumab	622,255,101
Trastuzumab	622,069,801, 622,069,901, 640,451,013, 620,001,938
Trastuzumab emtansine	622,264,401, 622,264,501
Trastuzumab deruxtecan	629,907,101
Atezolizumab	622,594,601
Pembrolizumab	622,515,701, 622,515,801
Hormonal treatment	
Tamoxifen citrate	620,001,885, 620,003,572, 620,003,573, 620,003,593, 620,003,594, 620,920,504, 620,921,003, 620,921,005, 620,921,201, 620,921,501, 620,921,701, 620,921,903, 620,921,905, 622,041,701, 622,053,001, 622,075,101, 622,317,900, 622,671,201, 622,671,301
Toremifene citrate	610,407,022, 610,407,023, 620,004,006, 622,169,001, 622,742,600, 622,742,700
Fulvestrant	622,101,401
Exemestane	610,462,026, 622,115,801, 622,118,801, 622,158,301
Letrozole	620,003,467, 622,411,401, 622,412,801, 622,413,201, 622,417,401, 622,418,401, 622,418,402, 622,420,001, 622,422,101, 622,427,401, 622,427,901, 622,429,201, 622,429,901, 622,431,001, 622,432,001, 622,433,901, 622,435,201, 622,436,701, 622,438,901, 622,475,600
Anastrozole	620,003,507, 622,180,501, 622,192,601, 622,195,001, 622,195,501, 622,198,501, 622,202,701, 622,204,401, 622,208,401, 622,208,701, 622,211,201, 622,213,401, 622,213,701, 622,215,501, 622,218,301, 622,220,301, 622,222,601, 622,222,701, 622,238,501, 622,309,400, 622,671,101, 622,689,100
Leuprorelin acetate	620,555,101, 620,555,201, 620,555,301, 620,555,401, 621,495,301, 622,266,501, 622,266,601, 622,298,301, 622,298,401, 622,444,901, 640,406,224, 640,432,015 640,432,016, 640,462,036, 642,490,119
Goserelin acetate	640,443,027, 640,462,004, 642,490,105
Medroxyprogesterone acetate	610,412,174, 610,433,100, 610,433,122, 610,454,075, 610,454,076, 612,470,030, 620,008,693, 620,537,802, 620,537,901, 620,538,001, 620,538,201, 620,538,401, 621,285,301, 622,736,700
Palbociclib	622,703,401, 622,703,501, 622,586,501, 622,586,601
Abemaciclib	622,653,801, 622,653,901, 622,654,001
Everolimus	621,980,901, 622,216,801, 622,226,301, 622,226,401
Methyltestosterone	610,407,122, 610,441,033, 612,460,005, 620,006,565
Mepitiostane	620,006,975
Ethinylestradiol	612,470,008, 620,009,249

As for disease name, we omitted suspected cases and only used confirmed cases. Nevertheless, the disease name in national health insurance claims data does not accurately match the diagnosed disease name. For example, in some cases, the disease name can continue to be displayed even when the patient is not undergoing any breast cancer treatment or it can be simply entered for medical fee claims. Therefore, during the study period, the claims data could have three groups of patients with breast cancer as the disease name: the group with breast cancer that received treatment for it, the group with breast cancer that received no treatment for it (e.g., older patients and patients with multiple comorbidities), and the group without breast cancer. Hence, patients with breast cancer as the disease name and a code for the specific breast cancer treatment were determined to have breast cancer. However, we excluded patients with a code for specific breast cancer treatment between May and July 2013 in their claim because their treatment could have started before the study period. Given that the confirmation or modification of the disease name may take some time, we included patients with breast cancer as the disease name up to June 2016. As for the age limit, national health insurance does not cover people aged 75 years or older. Therefore, we included only those patients who had breast cancer and were aged less than 70 at the start of treatment, which allowed them to be followed up after 5 years.

### Grouping patients by breast cancer treatment stage

We categorized patients with breast cancer into three groups based on the specific breast cancer treatment conducted during the study period: surgical treatment without axillary lymph node dissection (ALND; surgery without ALND group), other radical treatment with or without postoperative adjuvant chemotherapy (other radical treatment group), and palliative therapy (palliative group). The treatment in these groups corresponded to the respective treatment recommended for breast cancer classified as Stage 0/I, Stage II/III, and Stage IV according to the Classification of Breast Carcinoma by the Japanese Breast Cancer Society [14]. Radical treatment was a treatment that included the surgical resection of the primary lesion. Patients who underwent radical chemoradiotherapy were included in the other radical treatment group. Palliative treatment included chemotherapy for a more than a year, molecular targeted therapy, and treatment for distant metastases, including the radiation of metastatic lesions.

Patients with breast cancer who underwent tumorectomy, mastectomy, and other radical treatment (i.e., the surgery without ALND and other radical treatment groups) were defined as the curable group, while those in the palliative group were defined as the non-curable group. Patients with breast cancer were further classified according to their HER2 and hormone receptor status based on the specific breast cancer treatment they received during the study period and by referring to the diagnosis and pharmaceutical codes [15]. At least two experts from the working group reviewed individual claims data. Any discrepancies were resolved through expert discussions and we confirmed the accuracy of the classification based on the patient’s breast cancer treatment.

### Calculating the outcome measures

The primary outcome was the total medical care costs incurred by the curable and non-curable groups over the 5 years following the initial specific breast cancer treatment. Owing to the recording of multiple disease names within a claim, we could not accurately determine the costs associated with individual diseases [16]. Consequently, we aggregated the total medical care costs for managing breast cancer, which comprised the costs related to diagnostic procedures, perioperative management, handling of surgery-related complications, management of chemotherapy-induced side effects, and treatment for symptoms associated with cancer progression, particularly in the terminal phase. The median survival time reported for metastatic breast cancer is approximately 30 months [17]. Although breast cancer holds potential for long-term recurrence [18, 19], one of the indications for the treatment of hormone-positive and follow-up for HER2-positive breast cancer is five years [20, 21]. As such, the study period was set to five years. Total medical care costs were calculated by summing the claimed amounts each month, rounded to the nearest thousand.

Four secondary outcomes were identified: (1) the medical care costs of specific breast cancer treatment for the curable and non-curable groups over the five years; (2) medical care costs (at six-month intervals for the first year and then yearly for the subsequent four years) for the three groups; (3) medical care costs for different age groups (20–29, 30–39, 40–49, 50–59, and 60–69 years) for the three groups; and (4) medical care costs based on hormone and anti-HER2 therapies. The medical care costs of specific breast cancer treatment were determined using the national fee schedule for 2021 in Japan and by referring to the codes in Table 1. The incidence rate of breast cancer was calculated based on the number of patients identified with breast cancer by our algorithm or those identified in the claims data.

### Statistical analysis

Continuous variables were described using means and standard deviations (SDs). Categorical variables were described using numbers. Medical care costs were calculated and displayed as mean (SD) and median (interquartile range, IQR). Patients with missing data were excluded from the analysis. These analyses were conducted using JMP Pro® 16.1.0 (SAS Institute Inc., Cary, NC, USA).

## Results

### Patients

**Fig. 1:**
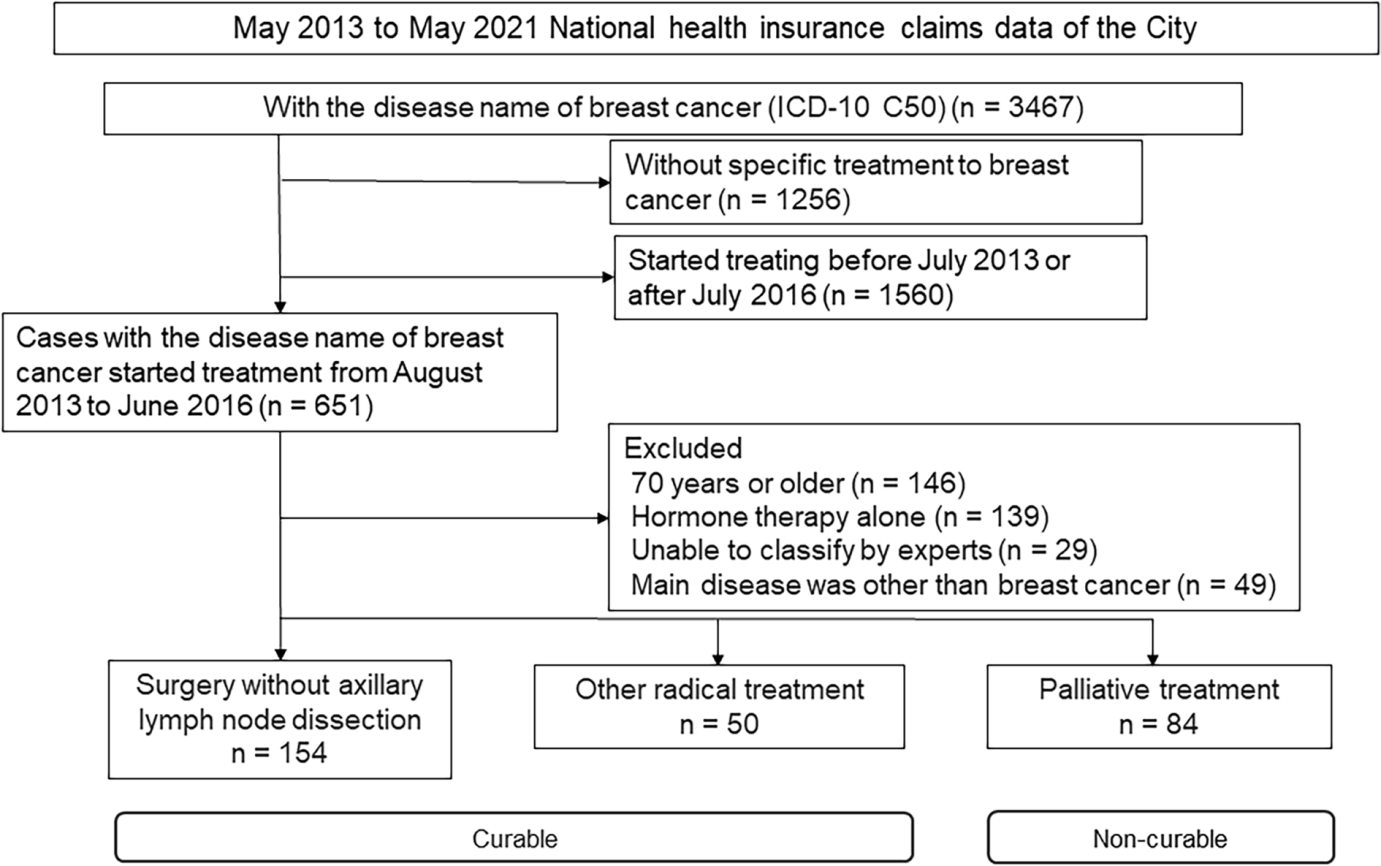
Flowchart showing the details of patient enrollment

In Hachioji City, national health insurance covered 61,368 women in 2021, representing 26.3% of the female population aged under 75 years. Between May 2013 and June 2016, we identified 3467 insurance claims associated with breast cancer diagnoses. Of these, 651 patients underwent a specific breast cancer treatment (see Table 1) between August 2013 and June 2016. We excluded patients aged 70 or older, those who underwent only hormone therapy, those who could not be classified into specific treatment groups, and those whose breast cancer treatment could have been administered for other diseases, including other malignancies (Fig. 1). This left 288 patients: 204 in the curable group (154 who underwent surgery without ALND and 50 who underwent other radical treatment) and 84 in the non-curable group. In the first round, the agreement rate for the group classification was 82%, but it reached 100% in the expert panel discussions. The mean ages of patients undergoing surgery without ALND, other radical treatment, and palliative treatment were 59.0, 60.6, and 58.0 years, respectively (Table 2). Table 3 shows the patient counts stratified by the hormone and anti-HER2 therapies.

**Table 2:** Age distribution of the breast cancer treatment groups

	20–39 years	40–49 years	50–59 years	60–69 years	Total	Mean age (years)
Surgery without axillary lymph node dissection	5	25	28	96	154	59.0 (8.8)
Other radical treatment	4	5	5	36	50	60.6 (10.1)
Palliative treatment	5	11	16	52	84	58.0 (9.7)

**Table 3:** Number of patients stratified by the hormone and anti-HER2 therapies in the breast cancer treatment groups

	Hormone + , HER2-	Hormone + , HER2 +	Hormone-, HER2 +	Hormone-, HER2-
Surgery without axillary lymph node dissection	97	17	6	34
Other radical treatment	40	2	3	5
Palliative treatment	47	16	10	11

### Medical care costs

The mean total medical care costs for the curable and non-curable groups for the 5 years were JPY 3958 thousand (SD 2664) and JPY 8289 thousand (8482), respectively. Table 4 summarizes the total medical care costs of the three treatment groups. The mean total medical care costs increased for all the treatment groups over the 5 years, with surgery without ALND being the least expensive (increasing from JPY 1431 thousand in the first 6 months to JPY 3565 thousand over the 5 years). The costliest treatment was palliative treatment, with a mean cost of JPY 1598 thousand in the first 6 months and JPY 8289 thousand over the 5 years.

**Table 4:** Cumulative total medical care costs in the breast cancer treatment groups

	Total medical care costs for six months	Total medical care costs for one year	Total medical care costs for two years	Total medical care costs for three years	Total medical care costs for four years	Total medical care costs for five years
	Mean (SD) [1000 JPY]					
Surgery without axillary lymph node dissection	1431 (554)	1890 (934)	2466 (1382)	2909 (1720)	3284 (1937)	3565 (2120)
Other radical treatment	1822 (654)	2719 (1201)	3469 (1880)	4128 (2464)	4644 (3048)	5173 (3657)
Palliative treatment	1598 (1170)	2817 (2175)	4565 (4041)	6114 (5940)	7230 (7228)	8289 (8482)
	Median (IQR) [1000 JPY]					
Surgery without axillary lymph node dissection	1349 (1062,1726)	1644 (1232,2231)	2108 (1574,2744)	2442 (1735,3273)	2707 (1928,4027)	3003 (2047,4362)
Other radical treatment	1889 (1321,2128)	2492 (1763,3658)	2989 (2156,4271)	3453 (2526,5362)	3955 (2668,5921)	4564 (2795,6636)
Palliative treatment	1306 (658,2312)	2119 (1112,4113)	2964 (1466,6396)	3879 (2307,7626)	4840 (2730,8586)	5374 (2947,10,578)

The mean medical care costs for specific breast cancer treatment for the curable and non-curable groups were JPY 1142 thousand (728) and JPY 3651 thousand (5337), respectively. This cost also increased for all the treatment groups over the 5 years. The costliest treatment was for metastasis/recurrence, increasing from JPY 1215 thousand in the first year to JPY 3651 thousand over the 5 years (Supplemental Table 1).

Table 5 displays total medical care costs for the different age groups. For most age groups, the most expensive treatment was other radical treatment with lymph node dissection. This was particularly expensive for the 30–39 age group (JPY 9791 thousand). In the non-curable group, medical care was expensive for older age groups (JPY 11,469 thousand for the 50–59 age group and JPY 8425 thousand for the 60–69 age group).

**Table 5:** Distribution of the total medical care costs for the five years by age group

	Mean (SD) [1000 JPY]					
	20–29 years	30–39 years	40–49 years	50–59 years	60–69 years	Total
Surgery without axillary lymph node dissection		2530 (730)	2375 (1128)	3228 (1741)	4027 (2315)	3565 (2120)
Other radical treatment		9791 (10,489)	5125 (3647)	4202 (1485)	4801 (2193)	5173 (3657)
Palliative treatment	4,489	4937 (1980)	4588 (4238)	11,469 (12,419)	8,425 (7,788)	8289 (8482)
	Median (IQR) [1000 JPY]					
Surgery without axillary lymph node dissection		2473 (1888–3199)	2339 (1574–3129)	2662 (2083–3924)	3309 (2244–5720)	3003 (2047–4362)
Other radical treatment		5243 (3449–20,683)	5721 (1527–8426)	4272 (2839–5529)	4564 (2723–6644)	4564 (2795–6636)
Palliative treatment	4,489	4909 (3035–6868)	2764 (1325–8864)	5968 (3694–17,427)	5684 (3361–11,276)	5374 (2947–10,578)

Table 6 shows medical care costs based on the hormone and anti-HER2 therapies. HER2 + , Hormone + patients had the highest mean total medical care cost over the 5 years, increasing from JPY 2257 thousand in the first 6 months to JPY 9906 thousand over the 5 years. Similarly, the HER2 + groups had a higher mean medical care cost for specific breast cancer treatment (Supplemental Table 2).

**Table 6:** Cumulative total medical care costs stratified by hormone therapy and anti-HER2 therapy

	Total medical care costs for six months	Total medical care costs for one year	Total medical care costs for two years	Total medical care costs for three years	Total medical care costs for four years	Total medical care costs for five years
	Mean (SD) [1000 JPY]					
HER2 + , Hormone-	2187 (1019)	3653 (1828)	5664 (4388)	7047 (6384)	8124 (8547)	9030 (10,439)
Hormone + , HER2-	1393 (651)	1934 (1082)	2645 (1717)	3323 (2454)	3894 (2995)	4406 (3537)
Hormone + , HER2 +	2257 (937)	4115 (1757)	6297 (3167)	7845 (5062)	8864 (6475)	9906 (7992)
Triple negative	1380 (789)	1885 (1364)	2439 (2475)	2958 (3779)	3283 (3990)	3497 (4015)
	Median (IQR) [1000 JPY]					
HER2 + , Hormone-	2109 (1325–2393)	3314 (2153–4744)	4027 (2489–7245)	5182 (3067–7245)	5313 (3067–7245)	5400 (3067–8243)
Hormone + , HER2-	1333 (955–1762)	1682 (1196–2316)	2256 (1582–2932)	2687 (1852–3838)	3048 (2084–4895)	3431 (2340–5699)
Hormone + , HER2 +	2215 (1753–2807)	3991 (3359–5262)	5333 (4568–6907)	5739 (4963–7710)	5939 (5367–9704)	6339 (5793–12,025)
Triple negative	1,261 (874–1759)	1640 (1064–2276)	1919 (1276–2754)	2372 (1527–3145)	2554 (1609–3611)	2636 (1744–3987)

## Discussion

Our findings revealed the treatment costs associated with different breast cancer treatment in a Japanese municipality. Total medical care costs increased over the 5 years for all types of breast cancer treatment. This result aligns with the findings of several previous studies: breast cancer-associated medical care costs rise over time due to factors such as disease progression, treatment complications, and the need for long-term supportive care [11]. We found that medical care costs are higher for the non-curable group than for the curable group, similar to the findings of a colorectal cancer study conducted in the same municipality [12]. This result highlights that medical care costs increase due to disease progression. Since we calculated medical care costs for the 5 years across medical institutions on a municipal basis, our findings will help municipalities and health insurers consider preventive measures and allocate resources for breast cancer screening programs.

Our findings also showed that patients in the HER2 + group had the highest mean total and treatment-specific medical care costs over the five years, suggesting that these patients may require more resource-intensive care. This finding aligns with that of a previous study that found that breast cancer subtype can significantly affect treatment costs [6]. Thus, molecular profiling stratifying patients into treatment groups could be crucial for examining the financial burden of breast cancer. We found that patients who received neither hormone nor HER2 treatment, suggestive of triple-negative breast cancer, did not have higher costs than those who received HER2 treatment. This may also be related to prognosis and breast cancer-specific costs because triple-negative breast cancer is known to have a poor prognosis [22, 23].

Further, we found that late-stage and HER2-positive breast cancer is associated with higher costs. While we were unable to analyze costs by both stage and subtype, early detection could be beneficial from the perspective of cancer screening. As some studies have reported, more expensive treatment such as HER2 treatment can improve patient outcomes [24–26], reinforcing the concept of value-based healthcare. Moreover, various antibody drugs other than HER2 treatment have been developed for treating breast cancer [27, 28]. Advances in therapeutic agents, while beneficial, can result in overtreatment. In light of this, clarifying medical care costs is essential for setting a strategic measure to curb such overtreatment as well as for minimizing the risk of recurrence.

This study has some limitations. First, data were lacking on patients’ income, socioeconomic status, and other potential confounding variables. Second, we only considered patients insured under Japan’s national health insurance, which may limit our findings’ generalizability. Third, no older adults were included because national health insurance does not insure people aged 75 or older. However, the mean age of the patients in all the groups hovered around the late 50 s, which is similar the findings of previous studies: the median age of breast cancer diagnosis is the late 50 s to early 60 s [29]. Future studies must consider medical care costs for older patients with breast cancer. Another important limitation was that this study was unable to capture the impact of emerging medical technologies and therapies. While some interventions such as BRCA genetic testing and treatment including immuno-checkpoint inhibitors and CDK4/6 inhibitors were approved before 2021, their actual use in clinical practice could be limited. Moreover, our data do not account for the impact of Oncotype DX for health insurance covered testing or the latest therapeutic regimens such as the introduction of immuno-checkpoint inhibitors and CDK4/6 inhibitors into perioperative care. Finally, as this study was conducted in one municipality, its findings are not generalizable to all municipalities or at the national level. Additionally, as this study was conducted on residents with national health insurance, the potential disparities between this insurance and others (e.g., employees’ health insurance) in terms of patient characteristics, treatment choices, and prognosis remain unexplored in the municipality. Similar studies should be conducted in other municipalities or at the national level to determine whether these trends hold universally or whether costs vary regionally. Despite these limitations, to the best of our knowledge, this is the largest study conducted in a municipal setting to examine the actual costs of breast cancer care.

Overall, this study shows that the early detection of breast cancer may reduce medical care at the patient level. This information could be instrumental in planning municipality-based health policies, resource allocation, and preventive strategies for breast cancer.

### Supplementary Information

Below is the link to the electronic supplementary material.

Supplementary file1 (DOCX 22 KB)

## Data Availability

This study was conducted under the city's regulations. All accessible data were available in the city's website as shown in ethical approval section.

## References

[1] Sung H, Ferlay J, Siegel RL (2021). Global cancer statistics 2020: GLOBOCAN Estimates of incidence and mortality worldwide for 36 cancers in 185 countries. CA Cancer J Clin.

[2] Lauby-Secretan B, Scoccianti C, Loomis D (2015). Breast-cancer screening—viewpoint of the IARC working Group. N Engl J Med.

[3] Ministry of Health Labor and Welfare, Japan. Cancer screening. Accessed July 6, 2023. https://www.mhlw.go.jp/stf/seisakunitsuite/bunya/0000059490.html

[4] Hubbard RA, Kerlikowske K, Flowers CI, Yankaskas BC, Zhu W, Miglioretti DL (2011). Cumulative probability of false-positive recall or biopsy recommendation after 10 years of screening mammography: a cohort study. Ann Intern Med.

[5] Hamashima C, on behalf of the Japanese Research Group for the Development of Breast Cancer Screening Guidelines, Hamashima C C, et al. The Japanese Guidelines for Breast Cancer Screening. Jpn J Clin Oncol. 2016;46(5):482–492. doi:10.1093/jjco/hyw008 10.1093/jjco/hyw008 27207993

[6] Brandão M, Morais S, Lopes-Conceição L (2020). Healthcare use and costs in early breast cancer: a patient-level data analysis according to stage and breast cancer subtype. ESMO Open.

[7] Shih YCT, Xu Y, Bradley C, Giordano SH, Yao J, Yabroff KR (2022). Costs around the first year of diagnosis for 4 common cancers among the privately insured. JNCI J Natl Cancer Inst.

[8] Watanabe T, Goto R, Yamamoto Y, Ichinose Y, Higashi T (2021). First-year healthcare resource utilization costs of five major cancers in Japan. Int J Environ Res Public Health.

[9] Molecular portraits of human breast tumours—PubMed. Accessed July 6, 2023. https://pubmed.ncbi.nlm.nih.gov/10963602/

[10] Harbeck N, Penault-Llorca F, Cortes J (2019). Breast cancer. Nat Rev Dis Primer.

[11] Patnaik JL, Byers T, Diguiseppi C, Denberg TD, Dabelea D (2011). The influence of comorbidities on overall survival among older women diagnosed with breast cancer. J Natl Cancer Inst.

[12] Utsumi T, Horimatsu T, Nishikawa Y (2021). Medical costs according to the stages of colorectal cancer: an analysis of health insurance claims in Hachioji. Japan J Gastroenterol.

[13] Research for Creating a Disease Name Determination Logic in National Database (NDB) Data Analysis. MHLW Grants System. Accessed August 7, 2023. https://mhlw-grants.niph.go.jp/project/27571

[14] Sakamoto G, Inaji H, Akiyama F (2005). General rules for clinical and pathological recording of breast cancer 2005. Breast Cancer Tokyo Jpn.

[15] Shimoi T, Nagai SE, Yoshinami T, et al. The Japanese Breast Cancer Society Clinical Practice Guidelines for systemic treatment of breast cancer, 2018 edition. Breast Cancer Tokyo Jpn. 2020;27(3):322–331. 10.1007/s12282-020-01085-0 10.1007/s12282-020-01085-0 PMC8062371 32240526

[16] Kimura S, Sato T, Ikeda S, Noda M, Nakayama T (2010). Development of a database of health insurance claims: standardization of disease classifications and anonymous record linkage. J Epidemiol.

[17] Valachis A, Carlqvist P, Ma Y (2022). Overall survival of patients with metastatic breast cancer in Sweden: a nationwide study. Br J Cancer.

[18] Cossetti RJD, Tyldesley SK, Speers CH, Zheng Y, Gelmon KA (2015). Comparison of breast cancer recurrence and outcome patterns between patients treated from 1986 to 1992 and from 2004 to 2008. J Clin Oncol Off J Am Soc Clin Oncol.

[19] Pedersen RN, Esen BÖ, Mellemkjær L (2022). The incidence of breast cancer recurrence 10–32 years after primary diagnosis. J Natl Cancer Inst.

[20] Pan H, Gray R, Braybrooke J (2017). 20-year risks of breast-cancer recurrence after stopping endocrine therapy at 5 years. N Engl J Med.

[21] Chumsri S, Li Z, Serie DJ (2019). Incidence of late relapses in patients with HER2-positive breast cancer receiving adjuvant trastuzumab: combined analysis of NCCTG N9831 (Alliance) and NRG oncology/NSABP B-31. J Clin Oncol Off J Am Soc Clin Oncol.

[22] Foulkes WD, Smith IE, Reis-Filho JS (2010). Triple-negative breast cancer. N Engl J Med.

[23] Bianchini G, Balko JM, Mayer IA, Sanders ME, Gianni L (2016). Triple-negative breast cancer: challenges and opportunities of a heterogeneous disease. Nat Rev Clin Oncol.

[24] Piccart-Gebhart MJ, Procter M, Leyland-Jones B (2005). Trastuzumab after adjuvant chemotherapy in HER2-positive breast cancer. N Engl J Med.

[25] Verma S, Miles D, Gianni L (2012). Trastuzumab emtansine for HER2-positive advanced breast cancer. N Engl J Med.

[26] Cortés J, Kim SB, Chung WP (2022). Trastuzumab deruxtecan versus trastuzumab emtansine for breast cancer. N Engl J Med.

[27] Robson M, Im SA, Senkus E (2017). Olaparib for metastatic breast cancer in patients with a germline BRCA mutation. N Engl J Med.

[28] Schmid P, Adams S, Rugo HS (2018). Atezolizumab and nab-paclitaxel in advanced triple-negative breast cancer. N Engl J Med.

[29] Giaquinto AN, Sung H, Miller KD (2022). Breast cancer statistics, 2022. CA Cancer J Clin.

