# Supplementary material for: Medical care costs according to the stage and subtype of breast cancer in a municipal setting: a case study of Hachioji City, Japan
Source: Breast Cancer. 2023 Nov 20;31(1):105–15. doi: 10.1007/s12282-023-01517-7 (PMC10764488; doi:10.1007/s12282-023-01517-7)
Supplement: Supplementary file 1 — Supplementary file1 (DOCX 22 KB) [file 12282_2023_1517_MOESM1_ESM.docx]

Supplemental Table 1. Cumulative breast cancer specific medical care costs in the breast cancer treatment groups.

|  | Total medical care costs for six months | Total medical care costs for one year | Total medical care costs for two years | Total medical care costs for three years | Total medical care costs for four years | Total medical care costs for five years |
| --- | --- | --- | --- | --- | --- | --- |
|  | Mean (SD) [1000 JPY] | | | | | |
| Surgery without axillary lymph node dissection | 613 (304) | 813 (502) | 938 (653) | 996 (680) | 1,044 (699) | 1,077 (715) |
| Other radical treatment | 732 (341) | 1,076 (586) | 1,209 (713) | 1,258 (717) | 1,303 (728) | 1,344 (739) |
| Palliative treatment | 668 (717) | 1,215 (1,353) | 1,900 (2,328) | 2,597 (3,474) | 3,072 (4,382) | 3,651 (5,337) |
|  | Median (IQR) [1000 JPY] | | | | | |
| Surgery without axillary lymph node dissection | 637 (329–790) | 752 (393–944) | 810 (499–1,039) | 832 (570–1,122) | 865 (602–1,206) | 885 (623–1,276) |
| Other radical treatment | 643 (495–923) | 882 (576–1,391) | 959 (629–1,612) | 1,004 (651–1,801) | 1,023 (688–1,803) | 1,122 (716–1,867) |
| Palliative treatment | 423 (193–883) | 770 (318–1,341) | 1,069 (422–2,194) | 1,264 (531–2,921) | 1,321 (617–2,994) | 1,460 (702–4,211) |

Supplemental Table 2. Cumulative breast cancer specific medical care costs stratified by hormone therapy and anti-HER2 therapy.

|  | Total medical care costs for six months | Total medical care costs for one year | Total medical care costs for two years | Total medical care costs for three years | Total medical care costs for four years | Total medical care costs for five years |
| --- | --- | --- | --- | --- | --- | --- |
|  | Mean (SD) [1000 JPY] | | | | | |
| HER2＋, Hormone- | 1,205 (788) | 2,139 (1,408) | 2,947 (2,272) | 3,538 (3,333) | 4,039 (4,618) | 4,553 (5,956) |
| Hormone＋, HER2- | 570 (335) | 750 (504) | 884 (640) | 1,025 (983) | 1,150 (1,379) | 1,284 (1,629) |
| Hormone＋, HER2+ | 915 (611) | 1,838 (1,116) | 2,881 (2,131) | 3,716 (3,617) | 4,142 (4,495) | 4,719 (5,649) |
| Triple negative | 545 (421) | 761 (765) | 899 (1,352) | 971 (1,517) | 1,014 (1,538) | 1,035 (1,552) |
|  | Median (IQR) [1000 JPY] | | | | | |
| HER2＋, Hormone- | 925 (675–1,594) | 1,671 (1,063–3,703) | 2,187 (1,063–4,477) | 2,386 (1,640–4,477) | 2,386 (1,640–4,477) | 2,386 (1,640–5,025) |
| Hormone＋, HER2- | 524 (320–771) | 728 (402–945) | 810 (504–1,067) | 865 (562–1,172) | 930 (623–1,287) | 986 (639–1,358) |
| Hormone＋, HER2+ | 810 (626–1,161) | 1,605 (1,273–2,265) | 2,540 (1,788–3,175) | 2,654 (1,956–3,324) | 2,696 (1,956–3,420) | 2,760 (2,071–3,917) |
| Triple negative | 432 (275–691) | 584 (303–853) | 620 (303–951) | 708 (321–1,021) | 708 (321–1,059) | 708 (321–1,059) |
